# The Medical College of Texas

**Published:** 1888-09

**Authors:** 


### THE MEDICAL COLLEGE OF TEXAS.

Prof. H. A. West, M. D., of the Texas Medical College Faculty, who went to New York and Philadelphia last month to purchase the chemical laboratory outfit, and other means of demonstration in physiology as well as chemistry, has returned, and we learn, has discharged the duty entrusted to him in a most satisfactory manner.'

The Texas Medical College (which, as before stated, is not the Medical Department of the University, but which is it thought will, in time, perhaps, develope into said department) is to be a first-class school of medicine, with a first-class equipment. The curriculum is to be as high as any in the land,—the course of instruction as thorough. This is a necessity ; for the school cannot succeed without the support of the profession of the State, and they will tolerate no half-way measures, no cheap education. The pride of the Faculty in their State, and the cordial sympathy and support of their colleagues in their laudable undertaking will stimulate them to deserve success ; and they will achieve it. Our best wishes attend their efforts.

